# Bone mineral density and trabecular bone score in adults with primary immunodeficiencies: a cross-sectional study

**DOI:** 10.3389/fendo.2026.1896132

**Published:** 2026-08-26

**Authors:** Anna Nowakowska-Płaza, Jakub Wroński, Arkadiusz Zegadło, Katarzyna Rybak, Agnieszka Rutkowska, Karina Jahnz-Różyk, Ewa Więsik-Szewczyk

**Affiliations:** 1 Department of Internal Medicine, Pneumonology, Allergology, Clinical Immunology and Rare Diseases, Military Institute of Medicine-National Research Institute, Warsaw, Poland; 2 Department of Rheumatology, National Institute of Geriatrics, Rheumatology and Rehabilitation, Warsaw, Poland; 3 Department of Radiology, Military Institute of Medicine-National Research Institute, Warsaw, Poland; 4 Department of Internal Medicine and Rheumatology, Military Institute of Medicine-National Research Institute, Warsaw, Poland

**Keywords:** appendicular lean mass, bone health, bronchiectasis, natural killer cells, osteoporosis, primary immunodeficiencies, trabecular bone score

## Abstract

**Introduction:**

Osteoporosis is increasingly recognized as an immune-mediated condition (“immunoporosis”), yet data on bone health in adults with primary immunodeficiencies (PIDs) remain limited. Chronic immune dysregulation and multisystem complications in PIDs may adversely affect skeletal integrity, but bone microarchitecture and muscle mass have not been comprehensively evaluated in this population. The aim of our study was to assess bone mineral density (BMD), trabecular bone score (TBS), and muscle mass in adults with PIDs. Exploratory analyses examined associations between these measures and clinical features and the main peripheral blood lymphocyte subsets.

**Methods:**

In this single-center cross-sectional study, 61 adult patients with PIDs underwent dual-energy X-ray absorptiometry (DXA) of the lumbar spine, total hip, femoral neck, and whole body. TBS was derived from lumbar spine DXA images, and the appendicular lean mass index was calculated to assess muscle mass. Clinical characteristics and immunological parameters, including peripheral blood lymphocyte subsets, were analyzed in relation to skeletal outcomes.

**Results:**

Based on age-appropriate DXA findings and clinical data, osteoporosis was diagnosed in 12 of 61 patients (19.7%). Three additional patients were diagnosed based on fragility fractures, increasing the overall prevalence to 15 of 61 (24.6%). Low BMD was more frequently detected at the hip than at the lumbar spine. Partially degraded or degraded TBS was observed in 15 of 61 patients (24.6%). Low muscle mass was identified in 17 of 61 patients (27.9%) and was associated with significantly lower BMD at all skeletal sites. Participants with bronchiectasis had lower femoral neck and total hip BMD and lower TBS, whereas those with enteropathy had lower BMD across all skeletal sites and lower TBS. Among participants eligible for T-score assessment (n = 15), NK-cell counts correlated positively with femoral neck (ρ = 0.63, p = 0.01) and total hip T-scores (ρ = 0.68, p < 0.01), and with TBS in the entire cohort (n = 61; ρ = 0.28, p = 0.03). CD4+, CD8+, and CD19+ cell counts were not associated with any evaluated densitometric parameters.

**Discussion and conclusions:**

In this cohort of adults with PIDs, reduced BMD and impaired TBS represented distinct, partially overlapping findings. Hip DXA appeared particularly informative, while TBS provided complementary information beyond BMD. These exploratory findings should be confirmed in prospective controlled studies.

## Introduction

Osteoporosis was traditionally defined by the World Health Organization (WHO) in 1993 as a systemic skeletal disorder characterized by reduced bone mass and microarchitectural deterioration, resulting in increased bone fragility and susceptibility to low-energy fractures, which represent its most clinically significant consequence (1, 2). Although postmenopausal and age-related osteoporosis are the most common forms, a wide range of chronic diseases are associated with an increased risk of osteoporosis, including autoimmune disorders and acquired immunodeficiencies such as human immunodeficiency virus (HIV) infection. In contrast, relatively little is known about the prevalence and characteristics of osteoporosis in patients with primary immunodeficiencies (PIDs).

PIDs are a clinically heterogeneous group of disorders caused by abnormalities in immune system development or function (3). According to the 2024 update of the International Union of Immunological Societies (IUIS) Expert Committee classification, PIDs result from more than 500 distinct genetic defects (4). However, in most adult patients, the underlying genetic cause remains unidentified. The most prevalent forms in adulthood include predominantly antibody deficiencies and immune dysregulation disorders. Advances in diagnostic approaches and treatment strategies—particularly immunoglobulin replacement therapy (IgRT)—have substantially improved survival and quality of life in this population. Consequently, adults with PIDs increasingly experience long-term complications related to chronic immune dysregulation, inflammation, accelerated biological aging, and prolonged exposure to immunomodulatory therapies. Osteoporosis may represent an important yet under-recognized comorbidity in this context.

Despite these considerations, bone health has received limited attention in adult PIDs populations. To date, only a few studies have addressed this issue, each involving relatively small cohorts (22–56 patients), and reporting a wide range of osteoporosis and osteopenia prevalence estimates (5–11). At the same time, patients with PIDs represent a unique human model of chronic immune dysregulation. Growing evidence indicates that osteoporosis is not merely a disorder of mineral metabolism but rather a disease of dysregulated bone remodeling in which the immune system plays a role (12, 13). This bidirectional interaction between immunity and skeletal homeostasis has given rise to the concept of “immunoporosis” (12).

Immune dysregulation, as observed in postmenopausal and senile osteoporosis, can profoundly influence bone metabolism by promoting osteoclast activation, altering osteoblast function, and sustaining inflammatory signaling pathways (13). Both innate and adaptive immune cells are involved in the regulation of bone turnover, including macrophages, dendritic cells, T and B lymphocytes, and natural killer (NK) cells. Depending on the microenvironmental context, immune cells may exert either pro-resorptive or bone-protective effects, highlighting the complexity of osteoimmunological interactions (14–21). M1 macrophages enhance bone resorption by promoting osteoclastogenesis, whereas M2 macrophages support bone formation by driving osteoblast differentiation (12, 16). Monocytes serve as osteoclast precursors, and neutrophils can stimulate bone resorption by upregulating membrane-bound RANKL (14, 15). NK cells appear to play a dual role: synovial NK cells from rheumatoid arthritis patients induce osteoclast formation via RANKL and M-CSF expression, yet IL-15–activated NK cells can inhibit osteoclastogenesis by killing osteoclasts through LFA-1– and DNAM-1–dependent mechanisms and by releasing anti-osteoclastogenic cytokines such as IFN-γ (16, 17). Similarly, B cells exert bidirectional effects on bone turnover (13). They may enhance osteoclastogenesis by providing RANKL, but also serve as a major source of osteoprotegerin (OPG). Their impact further depends on T-cell–derived signals: Th1 cytokines inhibit, whereas Th2 cytokines promote, osteoclast formation (18, 22). Among T-cell subsets, Th17 cells promote bone loss through increased RANKL and TNF-α expression, while regulatory T cells (Tregs) protect against bone resorption via secretion of IL-4, IL-10, and TGF-β1, and by suppressing Th17 responses and osteoclast differentiation (20, 21).

### Purpose

Given the limited evidence available in adults with PIDs, the present single-center study aimed to provide a comprehensive evaluation of bone health in a well-characterized cohort. Beyond standard assessment of bone mineral density (BMD), we incorporated trabecular bone score (TBS) to qualitatively assess trabecular microarchitecture and appendicular lean mass index (ALMI) to evaluate muscle mass (23). In addition, we investigated associations between skeletal parameters, main peripheral lymphocyte subsets (T, B, and NK cells), and PID-related clinical complications. By integrating immunological and densitometric data, this study seeks to extend the clinical understanding of immunoporosis and its relevance in adults with PIDs.

## Materials and methods

### Study population

This study included adult patients (≥18 years) with PIDs followed at the Outpatient Immunology Clinic of the Military Institute of Medicine – National Research Institute in Warsaw, Poland. All diagnoses were established by an experienced clinical immunologist in accordance with the European Society for Immunodeficiencies (ESID) registry criteria (24) and subsequently classified according to the 2024 IUIS Expert Committee classification (4).

Exclusion criteria were: (i) the presence of bilateral hip endoprostheses or spinal stabilization preventing reliable densitometric assessment; (ii) body mass index (BMI) <15 kg/m² or >35 kg/m², in line with manufacturer specifications and International Society for Clinical Densitometry (ISCD) recommendations indicating unreliable TBS measurements outside this range; (iii) inability to provide written informed consent, and (iv) established diagnosis of primary hypoparathyroidism, hyperthyroidism, type 1 diabetes, rheumatoid arthritis, osteogenesis imperfecta (v) pregnancy, and (vi) current or previous treatment with antiresorptive or anabolic medication for osteoporosis.

### BMD and body composition assessment

BMD was assessed using dual-energy X-ray absorptiometry (DXA) of the lumbar spine (L1–L4), total hip, femoral neck, and whole body. DXA measurements were performed using a single Lunar Prodigy GE densitometer (GE Healthcare; device ID DF + 510535). Daily calibration and quality-control procedures were performed before patient scanning using the manufacturer-provided calibration phantom. Calibration reports were reviewed and archived, and additional monthly phantom measurements were performed to assess long-term system stability. Scans were acquired by trained technologists using standardized patient positioning and acquisition procedures. All scans were reviewed for technical quality, and vertebrae or regions affected by severe degenerative changes, fractures, artifacts, or metal implants were excluded from the analysis. T-scores were derived from the National Health and Nutrition Examination Survey (NHANES) III reference database. Z-scores were derived from a reference database based on ethnicity (Germany), gender, and age.

Osteoporosis was defined according to the ISCD recommendations. In postmenopausal women and men aged ≥50 years, osteoporosis was diagnosed based on a T-score ≤−2.5 at the lumbar spine, total hip, or femoral neck alone. Osteopenia was defined as a T-score between -1.0 and -2.5. In premenopausal women and men aged <50 years, Z-scores were used, and a Z-score ≤−2.0 was reported as BMD below the expected range for age (25). In accordance with the ISCD position statement that allows a clinical diagnosis of osteoporosis in younger adults with secondary causes of low bone density or bone loss, PIDs were considered underlying chronic systemic conditions potentially associated with secondary bone loss (26). Therefore, all younger participants with a Z-score ≤−2.0 were classified as having osteoporosis in the context of PIDs. A clinical diagnosis was also made in participants with a low-energy fracture and osteopenia.

TBS, an indirect texture-based index related to trabecular microarchitecture, was calculated from lumbar spine DXA images using TBS iNsight software version 3.0.0.14. BMI-adjusted TBS values were generated automatically by the software according to the manufacturer’s recommendations for BMI of 15-35kg/m², (Medimaps Group); all patients were within this BMI range (27). TBS values were categorized as degraded (<1.20), partially degraded (1.20–1.349), or normal (≥1.35), in accordance with Silva et al. (28, 29) and the manufacturer’s recommendations. As these thresholds were derived primarily from older populations, their application to younger adults should be interpreted with caution.

Body composition was assessed using whole-body DXA. ALMI was calculated as the sum of lean mass in the upper and lower limbs divided by height squared (kg/m²). ALMI cut-off values indicating low muscle mass were: <5.5 kg/m² for women and <7.0 kg/m² for men (23).

### Laboratory evaluation

Laboratory assessment included measurements of serum 25-hydroxyvitamin D [25(OH)D], calcium, and phosphorus. Serum 25(OH)D concentrations were determined using enzyme-linked immunosorbent assay (ELISA) and classified as optimal (30–50 ng/mL), suboptimal (20–29 ng/mL), or deficient (<20 ng/mL). Laboratory screening for selected secondary causes of bone loss included serum calcium, phosphate, and creatinine with estimated glomerular filtration rate; fasting glucose; and thyroid-stimulating hormone, alanine aminotransferase, aspartate aminotransferase, gamma-glutamyl transpeptidase, and alkaline phosphatase. Parathyroid hormone was measured in participants with hypercalcemia.

All blood samples were drawn during routine visits. If patients were on immunoglobulin replacement therapy (IgRT), blood samples were drawn on the day before the IgG infusion, in accordance with national treatment reimbursement regulations.

Immunological standard evaluation comprised quantitative measurements of serum immunoglobulins IgG, IgA, and IgM, assessed by nephelometry (Siemens), and IgE, measured by fluoroimmunoassay using the Phadia 250 system (Thermo Fisher Scientific). Reference ranges were defined as follows: IgG 700–1600 mg/dL, IgA 70–400 mg/dL, IgM 40–230 mg/dL, and IgE 10–135 mg/dL.

Lymphocyte subset counts were retrieved from medical records. If these data were unavailable, the main lymphocyte subsets were assessed at study enrollment using whole-blood multiparameter flow cytometry, following previously described approaches (30). Absolute counts of the following lymphocyte subsets were included in the analysis: CD3+ T cells, CD4+ T cells, CD8+ T cells, CD19+ B cells, and CD3−CD16+CD56+ NK cells. The laboratory reference intervals were 900–2100 cells/µL for CD3+ T cells, 500–1300 cells/µL for CD4+ T cells, 280–900 cells/µL for CD8+ T cells, 120–400 cells/µL for CD19+ B cells, and 90–630 cells/µL for NK cells.

### Questionnaire and clinical data

Participants completed a structured questionnaire addressing lifestyle factors and clinical variables relevant to bone health. These included physical activity level, dietary calcium intake (with particular attention to dairy product consumption), smoking status, alcohol consumption, regular versus irregular vitamin D supplementation, family history of osteoporosis or hip fracture, chronic glucocorticoid (GC) therapy, history of low-energy fractures, and comorbid conditions independent of primary immunodeficiency. Chronic GC exposure was defined as a daily dose equivalent to ≥5 mg of prednisone administered for at least 3 months, either currently or at any time in the past. Detailed information on cumulative GC exposure was not available; however, a minimal cumulative dose of 450 mg of prednisone was reported according to the above-mentioned definition. In female patients, additional information regarding age at menopause and the presence of premature menopause (defined as onset before 45 years of age) was collected.

Clinical data were extracted from medical records and included age at PIDs diagnosis, disease duration, and the presence of PID-related complications. These comprised pulmonary involvement (including interstitial lung disease and bronchiectasis), enteropathy, lymphoproliferative disease or lymphoma, and cytopenias. Pulmonary involvement was confirmed by chest computed tomography and pulmonary function testing. Enteropathy was diagnosed based on compatible clinical symptoms combined with characteristic endoscopic and histopathological findings. Cytopenias were defined according to standard hematological criteria. Associations between individual osteoporosis risk factors and bone parameters were subsequently explored.

### Statistical analysis

Complete DXA and TBS data were available for all 61 participants, and no data imputation was required. The compliance of the data with the normal distribution was assessed using the Shapiro–Wilk test. The qualitative variables were presented as counts and percentages; quantitative variables with a normal distribution as mean ± standard deviation (SD); and quantitative variables without a normal distribution as median with interquartile range (IQR). The observed differences between groups were assessed with the Student’s t-test for variables with a normal distribution and the Mann-Whitney U test for variables without normal distribution. For categorical variables, the Chi-squared test or Fisher’s exact test for values less than 5 were used. The correlation was determined using the Pearson correlation coefficient for variables with a normal distribution and the Spearman rank correlation coefficient when normality assumptions were not met. Analyses of associations between skeletal parameters and clinical or immunological variables were exploratory and hypothesis-generating. Given the modest sample size and clinical heterogeneity of the cohort, no multivariable models were fitted, and no correction for multiple testing was applied. All reported p-values are unadjusted. Statistical analysis was performed using Statistica 13.3 software (StatSoft Polska, Kraków, Poland).

The study was approved by the Bioethics Committee of the Military Institute of Medicine – National Research Institute in Warsaw, Poland (approval no. 40/W/2025). Written informed consent was obtained from all participants, and all participants were covered by study-specific insurance.

## Results

### Study group characteristics

From 01.01.2025 to 30.07.2025, 65 consecutive adult patients (≥18 years) with PIDs were approached for participation in the study. Of these, 3 declined to participate, and 1 patient was excluded due to pregnancy. Ultimately, the study cohort comprised 61 patients with PIDs, including 24 women (39.3%; 18 premenopausal and 6 postmenopausal) and 37 men (60.7%; 28 aged <50 years and 9 aged ≥50 years). The median age at assessment was 39 years (IQR 31–48 years). The median age at diagnosis was 30 years (IQR 14–40 years).

According to the 2024 IUIS classification, patients were categorized into three main groups: combined immunodeficiencies (CID) with associated or syndromic features 6/61 (9.8%), predominantly antibody deficiencies 48/61 (78.7%), and diseases of immune dysregulation 7/61 (11.5%).

Among patients with predominantly antibody deficiencies, the most common diagnoses were common variable immunodeficiency (CVID), unclassified antibody deficiency, and X-linked agammaglobulinemia (XLA), accounting for 26/61 (42.6%), 11/61 (18.0%), and 8/61 (13.1%) cases, respectively.

The CID group included Ataxia telangiectasia 1/6 (16.6%), DiGeorge syndrome 1/6 (16.6%), Hyper IgE STAT3LOF syndrome 1/6 (16.6%), Nijmegen syndrome 1/6 (16.6%), and unclassified CID 2/6 (33.3%).

Detailed diagnostic characteristics and clinical data of the study population are presented in **Table 1**.

**Table 1:** Characteristics of 61 adult patients with PIDs.

Age, years (median, IQR)	39 (31-48)
Gender, female (n, %)	24 (39.3%)
BMI (mean, +/-SD)	25.2 (+/-4.3)
Smoking current (n, %)	8 (13.1%)
• Diagnosis (n, %) • CVID • Unclassified antibody deficiency • XLA • Agammaglobulinemia (AR n=1;genetically negative n=2) • ALPS • CTLA4 deficiency • STAT3 GOF • CID	26 (42.6%) 11 (18.0%) 8 (13.1%) 3 (5%) 3 (5%) 2 (3.3%) 2 (3.3%) 6 (9.8%)
IEI category according to IUIS (n, %) II Combined immunodeficiencies III Predominantly antibody deficiencies IV Diseases of immune dysregulation	6 (9.8%) 48 (78.7%) 7 (11.5%)
Age at diagnosis, years (median, IQR)	30 (14-40)
Disease duration, years (median, IQR)	8 (4-18)
Glucocorticoid chronic therapy (daily dose equivalent ≥5 mg of prednisone administered for at least 3 months) (n, %)	25 (41%)
Alcohol intake ≥ 3 units/day (n, %)	1 (1.6%)
At least moderate (3x30min/week) physical activity (n, %)	42 (68.9%)
A diet rich in calcium (n, %)	27 (44.2%)
Family history of hip fracture (n, %)	7 (11.5%)
Vitamin D supplementation (n, %)	42 (68.9%)
Immunoglobulin replacement therapy (n, %) - subcutaneous - intravenous	57 (93.4%) 56 (91.8%) 1 (1.6%)
Lung involvement (n, %) - interstitial lung disease, including GLILD - bronchiectasis	31 (50.8%) 7 (11.5%) 24 (39.3%)
Enteropathy (n, %)	11 (18%)
Polyclonal Lymphoproliferation (n, %)	22 (36.1%)
Autoimmune hemolytic anemia (n, %)	11 (18%)
Thrombocytopenia (n, %)	16 (26.2%)
Chronic kidney disease, GFR<60ml/min (n, %)	1 (1.6%)

### BMD and body composition assessment

**Figure 1:**
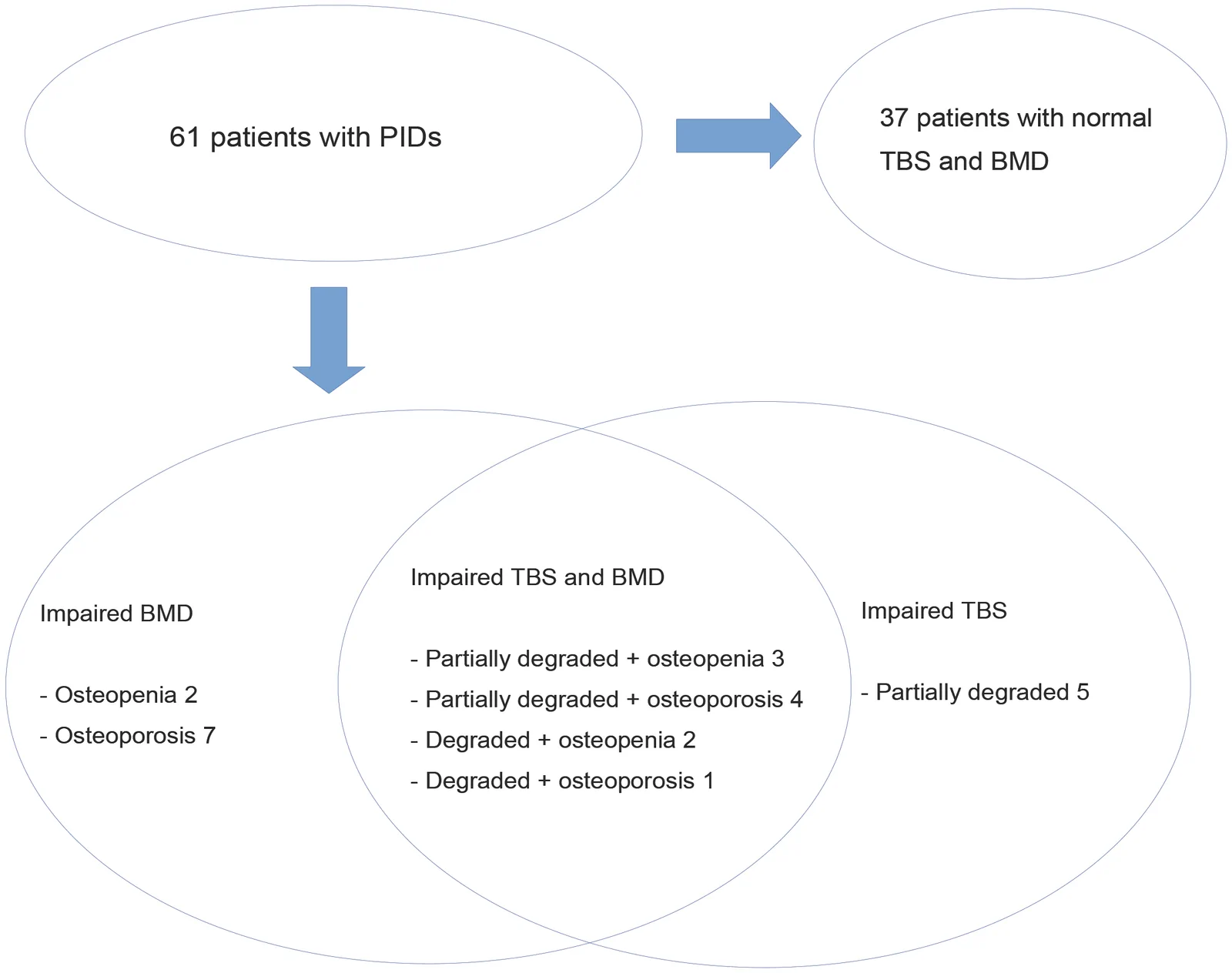
Bone-related DXA and TBS findings in 61 patients with primary immunodeficiencies (PIDs).

Overall, 24 (39.3%) patients had at least one abnormal bone-related parameter (BMD or TBS, **Figure 1**). Based on age-appropriate DXA findings and clinical context, osteoporosis was identified in 12 patients (19.7%), including 9 of 37 males (24.3%; all in men aged <50 years based on Z score in context of PIDs) and 3 of 24 females (12.5%; 2 postmenopausal based on T score and 1 premenopausal based on Z score in context of PID). Osteoporosis was detected by femoral DXA alone in 7 patients (58.3%), at both the femoral sites and lumbar spine in 2 patients (16.7%), and at the lumbar spine alone in 3 patients (25%).

One patient with DXA-confirmed osteoporosis sustained a low-energy humerus fracture. Three additional patients with osteopenia (1 male aged >50 years and 2 postmenopausal females) also experienced fragility fractures of ribs and vertebrae, resulting in a clinical diagnosis of osteoporosis. Overall, osteoporosis was diagnosed in 15 patients (10 of 37 males and 5 of 24 females). TBS did not differ significantly between patients with and without fractures.

Impaired trabecular bone microarchitecture, defined as partially degraded or degraded TBS, was observed in 15 patients (24.6%). Specifically, TBS was partially degraded in 12 patients (19.7%) and degraded in 3 patients (5.0%). Low muscle mass, defined by the appendicular lean mass index criteria, was present in 17 patients (27.9%).

The prevalence of osteoporosis, impaired bone microarchitecture, and low muscle mass is summarized in **Table 2**.

**Table 2:** Bone mineral density and microarchitecture, osteoporotic fractures, and low muscle mass among patients.

Bone mineral density (n, %) - Normal BMD - Osteopenia - Densitometric osteoporosis - Osteoporosis based on fragility fracture	42 (68.9%) 4 (6.6%) 12 (19.7%) 3 (4,9%)
Bone microarchitecture (n, %) - Normal - Partially degraded Degraded	46 (75.4%) 12 (19.7%) 3 (5%)
Vitamin D status (n, %) - Normal - Suboptimal - Deficiency	32 (52.5%) 18 (29.5%) 11 (18%)
Osteoporotic fractures (n, % F/M) Humerus: 22-year-old man with X-linked agammaglobulinemia Ribs: 41-year-old woman with STAT3 LOF hyper IgE syndrome Vertebrae: 52-year-old man with unclassified antibody deficiency, lymphoma, and enteropathy, and 69-year-old woman with unclassified antibody deficiency	4 (6.6%, 2/2)
Low muscle mass (n, %)	17 (27.9%)

Among classical osteoporosis risk factors, BMI correlated positively with BMD and T-scores at all measured skeletal sites, as well as with TBS. ALMI also showed positive correlations with BMD at all sites, total hip T-scores, and TBS (**Supplementary Table 1**).

Patients with low muscle mass had significantly lower BMD at the lumbar spine (1.11 vs. 1.22 g/cm², p = 0.009), total hip (0.90 vs. 1.02 g/cm², p = 0.002), and femoral neck (0.85 vs. 1.04 g/cm², p < 0.001), as well as lower T- and Z-scores at these sites. Differences in TBS between patients with and without low muscle mass did not reach statistical significance (p = 0.17).

No significant associations were observed between bone parameters and sex, age, disease duration, or age at PID diagnosis. Chronic GC therapy was not associated with BMD or TBS values.

### Clinical parameters specific to PIDs and bone health

BMD and body composition parameters were compared across the three main IUIS categories represented in the cohort. Patients with combined immunodeficiencies with associated or syndromic features exhibited significantly lower total hip BMD compared with those with predominantly antibody deficiencies (0.84 vs. 1.01 g/cm², p = 0.006).

Subsequently, associations between bone parameters and specific PID-related clinical manifestations were analyzed. Patients with bronchiectasis (n = 24) demonstrated significantly lower femoral neck BMD (0.92 vs. 1.03 g/cm², p = 0.006), femoral neck T-scores (–1.35 vs. –0.30, p = 0.034), total hip BMD (0.92 vs. 1.02 g/cm², p = 0.007), and TBS (1.37 vs. 1.43, p = 0.048) compared with patients without bronchiectasis.

Patients with enteropathy (n = 11) exhibited significantly reduced bone parameters across all assessed skeletal sites. These included lower lumbar spine BMD (1.08 vs. 1.21 g/cm², p = 0.010) and T-scores (–1.88 vs. –0.62, p = 0.001), femoral neck BMD (0.89 vs. 1.01 g/cm², p = 0.020) and T-scores (–1.56 vs. –0.30, p = 0.010), total hip BMD (0.90 vs. 1.00 g/cm², p = 0.028) and T-scores (–1.72 vs. 0.10, p = 0.002), as well as lower TBS values (1.32 vs. 1.42, p = 0.007).

Additionally, patients with lymphadenopathy (n=14) had significantly lower lumbar spine Z-scores than those without lymphadenopathy (–0.88 vs. –0.01; p = 0.006). Thrombocytopenia was associated with both reduced lumbar spine Z-scores (–0.84 vs. –0.13, p = 0.040) and lower TBS values (1.34 vs. 1.42, p = 0.010), indicating impaired bone microarchitecture.

No additional significant associations between bone parameters and other PID-related clinical manifestations were identified.

### Immunological parameters and bone parameters

First, correlations between peripheral blood lymphocyte subsets and bone parameters were assessed. Total CD3+ T-cell counts showed a moderate but statistically significant positive correlation with femoral neck T-scores (ρ =0.53, p=0.04). In contrast, no significant associations were observed between CD4+, CD8+, or CD19+ lymphocyte counts and densitometric parameters.

**Figure 2:**
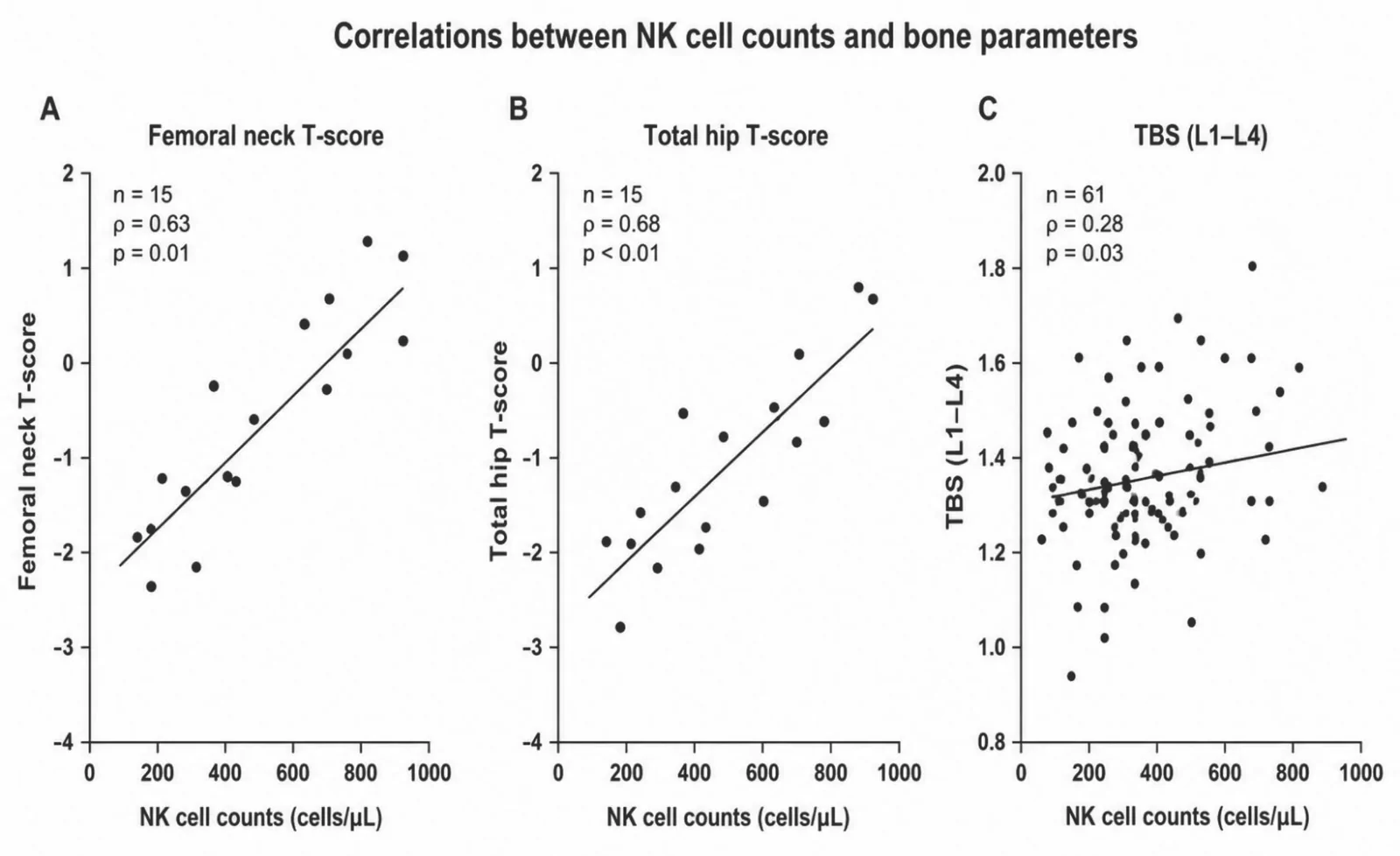
Spearman correlation of NK cell counts with TBS, T-score in the femoral neck, and total hip.

NK cell counts correlated positively with femoral neck (n=15, ρ =0.63, p=0.01) and total hip (n=15, ρ =0.68,p<0.01) T-scores, as well as with TBS (n=61, ρ =0.28, p=0.03) (**Figure 2**). No statistically significant correlations were observed between NK-cell counts and absolute BMD or age-appropriate Z-scores. Patients with reduced NK-cell counts exhibited significantly lower femoral neck T-scores (n=10, –1.43 vs. n=7, –0.10, p = 0.003) and total hip T-scores (n=10, –1.31 vs. n=7, 0.20, p = 0.010) compared with patients with NK-cell counts within the reference range.

Additionally, current serum IgG levels correlated positively with serum 25(OH)D concentrations. Patients with reduced IgG levels had significantly lower vitamin D levels than those with normal IgG concentrations (15.5 vs. 32.0 ng/mL, p = 0.014).

## Discussion

Osteoporosis in patients with PIDs remains an under-recognized complication, with only a limited number of small studies addressing this issue to date. In the present study, we analyzed a comparatively large and clinically representative cohort of adults (≥18 years) with PIDs managed in routine clinical practice. In this cohort, reduced BMD and impaired bone microarchitecture were commonly observed, with osteoporosis diagnosed in 24.6% of patients (in 12 patients based on densitometric criteria in the context of PIDs and in 3 due to fragility fractures). Previously published reports have shown varying prevalences of low BMD (9%-57.6%) in smaller populations of pediatric or adult patients with PID and based on different densitometric criteria (5–11). However, our study is unique in its comprehensive densitometric and clinical data and adds descriptive data on skeletal abnormalities in adults with PIDs (5–9, 11).

Current ISCD guidelines recommend BMD assessment in all women aged ≥65 years and men aged ≥70 years, as well as in younger individuals with established risk factors, including low body weight, prior fragility fractures, chronic GC use, or medical conditions associated with bone loss (25). Several chronic diseases—including diabetes, inflammatory rheumatic disorders, inflammatory bowel disease, celiac disease, chronic liver and kidney disease, hyperthyroidism, Cushing’s syndrome, and HIV infection—are well recognized as secondary causes of osteoporosis (31–36).

In this context, our findings suggest that PIDs may represent an additional condition associated with an increased risk of skeletal impairment. Notably, osteoporosis was particularly frequent among men in our cohort (27% vs 20.8% in females). This observation highlights the potential for underdiagnosis of osteoporosis in male patients with PIDs and suggests that bone health assessment should be considered in patients in this group. However, in the absence of a control group, our study cannot determine whether the prevalence of osteoporosis is higher than in the general population.

An additional noteworthy finding of the present study is the predominance of femoral DXA in detecting osteoporosis in adults with PIDs. In our cohort, osteoporosis was more frequently identified at the hip than at the lumbar spine, with femoral DXA alone accounting for the majority of densitometric diagnoses (58.3%). This pattern differs from the conventional expectation that trabecular-rich sites such as the lumbar spine are affected earlier, particularly in younger individuals. Traditionally, lumbar spine DXA is often favored in younger adults because trabecular bone deteriorates earlier than cortical bone (28, 29). The predominance of femoral abnormalities may indicate a site-specific pattern of skeletal involvement; however, the cross-sectional design does not permit conclusions regarding the temporal sequence of cortical and trabecular bone changes. Because vertebrae affected by compression fractures, marked degenerative changes, or other relevant artifacts were excluded from the analysis, these technical factors are unlikely to account for the more frequent detection of abnormalities at the femoral sites. The mechanisms underlying potential preferential impairment of cortical bone in PID remain unclear and warrant further study.

This study is also the first to assess TBS in patients with PID. TBS allows qualitative assessment of trabecular microarchitecture. Degraded or partially degraded microarchitecture was identified in 24.6% of patients. Lower TBS values indicate degraded bone structure and increased fracture risk. Although optimal cut-off values remain debated, both the ISCD and the ESCEO endorse TBS for fracture-risk assessment in postmenopausal women and men over 50 (28, 37). Reduced TBS has been reported across various secondary osteoporosis settings, including diabetes (38), primary hyperparathyroidism (39, 40), acromegaly (41), anorexia nervosa (42), chronic GC use (24), aromatase inhibitor therapy (43), rheumatic diseases (44, 45), kidney transplantation (46), and hemodialysis (47). Importantly, impaired TBS was observed in our cohort among patients with and without densitometric osteoporosis, indicating that alterations in trabecular microarchitecture may be present even in the absence of BMD-defined osteoporosis. This finding suggests that TBS may provide complementary information to BMD in assessing skeletal health in adults with PIDs. Although TBS did not differ significantly between patients with and without fractures in the present study, this observation should be interpreted with caution and may reflect limited statistical power.

Low muscle mass represents an additional musculoskeletal vulnerability. Based on ALMI, low muscle mass was present in 27.9% of our cohort and was associated with significantly lower BMD at all assessed skeletal sites, as well as lower T- and Z-scores. These findings suggest a relationship between reduced muscle mass and impaired bone parameters in patients with PIDs.

Among standard fracture risk factors, as in Mohebbi et al. (7), we observed that BMD correlated positively with BMI. Unfortunately, standard risk calculators such as FRAX^®^ are not validated for individuals under 40 years of age (48, 49), limiting their utility in many patients with PID. Interestingly, chronic GC therapy—commonly implicated in secondary osteoporosis—was not associated with BMD or TBS in our cohort. However, detailed cumulative GC exposure could not be assessed, which may have limited our ability to evaluate its association with bone parameters. Identifying disease-specific osteoporosis risk factors is therefore essential. Differences in total hip BMD were observed between diagnostic groups; patients with combined immunodeficiencies with associated or syndromic features had significantly lower total hip BMD than those with predominantly antibody deficiencies. Whether these reflect differences in the underlying disease phenotype, as has been suggested for patients with hyper IgE syndrome (10), requires further investigation. PID-related comorbidities were also associated with selected skeletal parameters. Our findings support a strong association between bronchiectasis and low BMD, consistent with other studies (5, 6). Additionally, enteropathy was associated with lower BMD across all measured skeletal sites and TBS values. We must underscore that enteropathy is a rare complication but severely impacts morbidity and mortality in patients with PIDs. Lymphadenopathy and thrombocytopenia were associated with lower lumbar spine Z-scores; intriguingly, thrombocytopenia also correlated with reduced TBS. Finally, participants with fractures had lower total hip BMD, whereas TBS did not differ between groups; however, the study was not designed to assess the predictive performance of either measure.

Data on vitamin D metabolism in PID remains limited. Previous reports identified a deficiency in 25–52% of patients (50, 51). In our cohort, deficiency was observed in 18% of patients, and vitamin D levels correlated positively with IgG levels. Because 70% of participants reported taking vitamin D supplements, this association may reflect greater adherence among individuals engaged in regular IgRT.

Our cohort was characterized by marked impairment of adaptive immune function. Previous studies have suggested associations between reduced B-cell counts and BMD, and between CD4+ T-cell counts and skeletal parameters in patients with PIDs (5, 6). In the present study, total CD3+ T-cell counts were positively associated with femoral neck T-scores, whereas no significant associations were observed for CD4+, CD8+, or CD19+ lymphocyte subsets.

In contrast, one of the most notable findings of our study was an exploratory association between NK-cell counts and selected densitometric parameters (femoral neck and total hip T-scores and TBS). Although NK cells have traditionally been implicated in osteoclast activation and bone resorption, increasing evidence indicates that their effects on bone metabolism are highly context-dependent and influenced by the local cytokine milieu and immune activation status (14). In this context, our findings suggest that variations in NK-cell counts may accompany differences in bone density and microarchitecture in adults with PIDs. Further studies incorporating detailed immunophenotyping and functional analyses are needed to clarify the role of NK cells in immune–bone interactions in this population.

Our study has several limitations. First, due to its cross-sectional design, causal inferences cannot be drawn. Second, the cohort comprised a heterogeneous group of PIDs (IUIS groups II–IV); however, 93.4% of patients required IgRT due to significant antibody deficiency. Some lymphocyte subset counts were retrospectively retrieved from immunophenotyping performed as part of the diagnostic work-up supporting the PIDs diagnosis and were therefore not contemporaneous with DXA assessment. This temporal difference may have affected the observed immune–bone associations. However, there is currently no consensus on the systematic serial assessment of lymphocyte subsets in patients with PIDs. The analyses focused on basic T-, B-, and NK-cell subsets that are routinely measurable, including in non-reference centers.

Third, we did not assess sarcopenia, as only muscle mass was evaluated using densitometric parameters, whereas muscle strength and physical performance, assessed using measures such as handgrip strength, gait speed, or the Timed Up and Go test, were not evaluated. Fourth, currently available TBS thresholds have been established primarily in older populations, and their interpretation in younger adults with primary immunodeficiencies should be approached cautiously. Finally, multiple exploratory comparisons were performed without correction for multiple testing, increasing the risk of false-positive findings. The modest sample size and clinical heterogeneity of the cohort limited the feasibility and reliability of multivariable adjustment for potentially relevant confounders. Consequently, the reported associations are unadjusted, may be affected by residual confounding, should not be interpreted as independent or causal, and require confirmation in larger studies.

Although relatively large for a rare disease, the sample size remains modest compared with typical osteoporosis studies and did not allow for multivariate analyses. Finally, the absence of a healthy control group limits direct comparative interpretation of the findings.

Nevertheless, the study has several notable strengths, including the use of TBS, detailed clinical phenotyping, and comprehensive densitometric evaluation. Importantly, we focused on immunological parameters and clinical assessments readily available in routine clinical practice rather than restricted to specialized immunology referral centers, thereby enhancing the potential applicability of our findings across medical specialties. To date, this study represents one of the most comprehensive assessments of bone health parameters in adult patients with PIDs.

## Conclusions

To date, PIDs have not traditionally been considered contributors to osteoporosis, despite increasing recognition of immune–bone interactions. In this cohort of adults with PIDs, reduced BMD, impaired TBS, and low muscle mass were commonly observed. Associations with bronchiectasis, enteropathy, disease phenotype, and NK-cell counts were exploratory and unadjusted and require confirmation in larger controlled studies. Femoral abnormalities were identified more frequently than lumbar spine abnormalities. Prospective studies with appropriately matched control groups are needed to assess temporal relationships, determine whether the prevalence of osteoporosis is increased in patients with PIDs, and inform evidence-based screening strategies.

## Data Availability

The original contributions presented in the study are included in the article/**Supplementary Material**. Further inquiries can be directed to the corresponding authors.
