# Supplementary material for: Bone mineral density and trabecular bone score in adults with primary immunodeficiencies: a cross-sectional study
Source: Front Endocrinol (Lausanne). 2026 Aug 26;17:1896132. doi: 10.3389/fendo.2026.1896132 (PMC13553275; doi:10.3389/fendo.2026.1896132)
Supplement: Supplementary file 1 [file DataSheet1.pdf]

| | BMI | ALMI |
|---|---|---|
| BMD L1-4 | ρ = 0.44 , p <0.01 | ρ = 0.28 , p = 0.01 |
| Z-score L1-4 | ρ = 0.81, p <0.01 | ρ = 0.45, p = 0.01 |
| T-score L1-4 | ρ = 0.01, p =0.12 | ρ = -0.06, p = 0.51 |
| BMD femoral neck | ρ = 0.38, p <0.01 | ρ = 0.36, p < 0.01 |
| Z-score femoral neck | ρ = 0.75, p <0.01 | ρ = 0.47, p = 0.08 |
| T-score femoral neck | ρ = 0.25, p = 0.09 | ρ = 0.13, p = 0.37 |
| BMD total hip | ρ = 0.49, p <0.01 | ρ = 0.48, p < 0.01 |
| Z-score total hip | ρ = 0.92, p <0.01 | ρ = 0.71, p < 0.01 |
| T-score total hip | ρ = 0.32, p = 0.03 | ρ = 0.23, p = 0.13 |
| TBS L1-4 | ρ = 0.31, p = 0.02 | ρ = 0.27, p = 0.03 |

*Tabela 1: Suppl Table 1. Correlations between Body Mass Index (BMI) and Appendicular Lean Mass Index (ALMI) and bone-related parameters*
